# Peroxidase-Generated Apoplastic ROS Impair Cuticle Integrity and Contribute to DAMP-Elicited Defenses

**DOI:** 10.3389/fpls.2016.01945

**Published:** 2016-12-23

**Authors:** Mantas Survila, Pär R. Davidsson, Ville Pennanen, Tarja Kariola, Martin Broberg, Nina Sipari, Pekka Heino, Erkki T. Palva

**Affiliations:** Division of Genetics, Viikki Plant Science Centre, Department of Biosciences, Faculty of Biological and Environmental Sciences, University of HelsinkiHelsinki, Finland

**Keywords:** *Arabidopsis*, cutin biosynthesis, necrotrophs, oligogalacturonides (OGs), Peroxidase 57 (PER57), reactive oxygen species (ROS)

## Abstract

Cuticular defects trigger a battery of reactions including enhanced reactive oxygen species (ROS) production and resistance to necrotrophic pathogens. However, the source of ROS generated by such impaired cuticles has remained elusive. Here, we report the characterization of *Arabidopsis thaliana ohy1* mutant, a *Peroxidase 57* (*PER57*) – overexpressing line that demonstrates enhanced defense responses that result both from increased accumulation of ROS and permeability of the leaf cuticle. The *ohy1* mutant was identified in a screen of *A. thaliana* seedlings for oligogalacturonides (OGs) insensitive/hypersensitive mutants that exhibit altered growth retardation in response to exogenous OGs. Mutants impaired in OG sensitivity were analyzed for disease resistance/susceptibility to the necrotrophic phytopathogens *Botrytis cinerea* and *Pectobacterium carotovorum*. In the *ohy1* line, the hypersensitivity to OGs was associated with resistance to the tested pathogens. This *PER57* overexpressing line exhibited a significantly more permeable leaf cuticle than wild-type plants and this phenotype could be recapitulated by overexpressing other class III peroxidases. Such peroxidase overexpression was accompanied by the suppressed expression of cutin biosynthesis genes and the enhanced expression of genes associated with OG-signaling. Application of ABA completely removed ROS, restored the expression of genes associated with cuticle biosynthesis and led to decreased permeability of the leaf cuticle, and finally, abolished immunity to *B. cinerea*. Our work demonstrates that increased peroxidase activity increases permeability of the leaf cuticle. The loss of cuticle integrity primes plant defenses to necrotrophic pathogens via the activation of DAMP-responses.

## Introduction

To fight off pathogens, animals depend on mobile defender cells and the adaptive immune system, while plants rely on the innate immunity of individual cells. In plants, these systems are activated by cell surface or intracellular receptors (Jones and Dangl, 2006). Cell surface pattern-recognition receptors (PRRs) recognize microbes via perception of pathogen-associated molecular patterns (PAMPs) (Bittel and Robatzek, 2007; Zipfel, 2008, 2009). PAMPs are typically conserved molecules characteristic to a whole class of microbes and include for example fungal chitin and microbe-derived structures like bacterial flagellin, EF-Tu or their peptide surrogates flg22 and elf18, respectively (Jones and Dangl, 2006; Zipfel, 2008). PAMPs can also be associated with non-pathogens and referred to as microbe-associated molecular patterns (MAMPs) (Zipfel, 2008). In addition to recognition of PAMPs, plants have the ability to recognize modified-self, including damage-associated molecular patterns (DAMPs) (Ferrari et al., 2013). A major category of DAMPs are plant cell-wall fragments released by the action of plant cell-wall degrading enzymes (PCWDEs) secreted by necrotrophic and hemibiotrophic pathogens. Pectin is a central component in plant cell walls and constitute a major target for PCWDEs during pathogen invasion. Oligogalacturonide (OG) fragments of pectin released by the action of pectin degrading enzymes such as polygalacturonases (PGs) are the best characterized plant DAMPs that activate innate immune responses (Ferrari et al., 2013; Bellincampi et al., 2014). The recognition of PAMPs and DAMPs leads to the activation of plant defenses and pattern-triggered immunity (PTI), which provide resistance to most non-adapted pathogens in a phenomenon called non-host resistance (Zipfel, 2009, 2014). Plants also rely on effector-triggered immunity (ETI). This type of perception involves intracellular receptors that directly or indirectly recognize pathogen effectors (Zipfel, 2014). According to current knowledge, recognition of PAMPs, DAMPs and effectors triggers overlapping signaling responses in the plant and indicate a difference in the speed, persistence and robustness rather than the quality of response between PTI and ETI (Espinosa and Alfano, 2004; Tsuda and Katagiri, 2010).

Among the responses downstream of PTI and ETI, oxidative burst is one of the earliest (Torres et al., 2006; Torres, 2010). The oxidative compounds, which can be detected after pathogen recognition, are collectively termed reactive oxygen species (ROS). ROS include superoxide, hydrogen peroxide, hydroxyl radical, singlet oxygen and nitric oxide. During most abiotic and biotic stress responses, plasma membrane-localized NADPH/NADH oxidases and cell wall-localized class III (CIII) apoplastic peroxidases are the major sources of ROS (Grant et al., 2000; Bolwell et al., 2002).

A family of 10 respiratory burst oxidase homolog (*RBOH*) genes has been identified in *A. thaliana* (Torres and Dangl, 2005). Their role in plant defense against a wide range of pathogens has been documented (Marino et al., 2012), however, only *RBOHD* appears to respond to PTI triggers (Morales et al., 2016). The *RBOHD*-dependent oxidative burst triggered by OG elicitors was reported to protect *Arabidopsis thaliana* plants from *Botrytis cinerea* (Galletti et al., 2008; L’Haridon et al., 2011). Despite the loss of the OG-elicited ROS-burst, *atrbohD* and *atrbohD/F* mutants still demonstrated ROS production in response to wounding and *B. cinerea*, as well as enhanced expression of defense-related genes, suggesting the involvement of additional sources of ROS.

CIII apoplastic peroxidases are encoded by a family of 73 genes in *A. thaliana* (Welinder et al., 2002). CIII peroxidase-generated oxidative bursts appear to play an important role in *A. thaliana* PTI. The accumulation of ROS, callose deposition and the activation of defense-related gene expression following treatment with bacterial flagellin were impaired in the *per33/per34* double mutant (Daudi et al., 2012). Pharmacological inhibitor-based studies have validated the notion that CIII peroxidases are key components of ROS production pathways during defense response (Soylu et al., 2005). Consistent with these results, exposure to pathogens was also observed to initiate an increase in the activity of CIII peroxidases (Lehtonen et al., 2009).

Chassot et al. (2008) demonstrated that mutants impaired in cuticle permeability are directly linked to enhanced resistance to necrotrophic pathogens. These cuticular mutants were found to constitutively produce ROS (L’Haridon et al., 2011). Importantly, cuticular mutants displayed enhanced expression of several CIII peroxidases, and the overexpression of these peroxidases provided resistance to *B. cinerea* (Chassot et al., 2008). Cuticle-derived resistance to *B. cinerea* was shown to be independent of the major plant defense signaling pathways mediated by salicylic acid (SA), jasmonic acid (JA), and ethylene (ET) (Chassot et al., 2008). In addition, *A. thaliana* and tomato mutants that displayed impaired abscisic acid (ABA) biosynthesis also exhibited constitutive accumulation of ROS, an increase in the permeability of the leaf cuticle and enhanced resistance to *B. cinerea* (Asselbergh et al., 2007; L’Haridon et al., 2011), suggesting the involvement of ABA signaling in cuticle formation. Perturbations in ABA signaling has been shown to lead to enhanced pathogen susceptibility in several plant systems (McDonald and Cahill, 1999; Audenaert et al., 2002; Mohr and Cahill, 2003). However, negative or positive role of ABA in disease resistance depends on the type of pathogen and it has been shown to modulate immune responses through ROS generation, defense gene expression, cuticle permeability and callose accumulation (Asselbergh et al., 2007; L’Haridon et al., 2011; Robert-Seilaniantz et al., 2011). For example, elevated levels of ABA negatively affect the defense against soil-born fungus *F. oxysporum*, by having an antagonistic effect on the JA-ET signaling network (Anderson et al., 2004). On the other hand, *aba1, aba2*, and *abi4-1* mutants were more susceptible to biotrophic *Pythium irregulare* and *Altenaria solani* pathogens highlighting the different roles of ABA in resistance to necrotrophic and biotrophic pathogens (Adie et al., 2007).

In this study, we explore the sources of ROS that are associated with increased cuticular permeability observed in an OG hypersensitive mutant. We show that cuticle permeability is impaired in plants overexpressing CIII peroxidases. Phenotypically, transgenic lines overexpressing CIII peroxidases are similar to cuticular mutants displaying impaired cuticle biosynthesis. In accordance, increased peroxidase activity was found to have a negative effect on the expression of cutin-biosynthetic genes. Importantly, in the presence of exogenously applied ABA, we observed the complete removal of ROS in all of the tested lines that otherwise exhibited an excessive accumulation of ROS. The ABA-triggered removal of ROS restored the expression of cutin-biosynthetic genes and ultimately also the permeability of the cuticle to a level similar to that observed in wild-type plants. Finally, we demonstrate that peroxidase-generated ROS increase cuticle permeability that prime plants to initiate enhanced defenses against necrotrophic pathogens by up-regulating the expression of OG-responsive defense-related genes.

## Materials and Methods

### Plant Material and Growth Conditions

*Arabidopsis thaliana* seeds were sown on a 2:1 mixture of vermiculite:peat (Finnpeat B2, Kekkilä Oyj, Tuusula, Finland) and stratified at 4°C for 2 days before being transferred to a growth chamber where they were grown at 23°C and a 12 h photoperiod with a light intensity of 280 μmol m^-2^ s^-1^ and 50–60% relative humidity. The following ecotypes and mutants were used: Columbia-0 (Col-0), C24, *gin1-3 (aba2)*, and *per57-1* (GK_325E06) were obtained from the Nottingham *A. thaliana* Stock Centre. The *pyr1pyl1pyl2pyl4pyl5pyl*8 (*112458*) sextuple mutant was obtained from Pedro L. Rodriguez, Instituto de Biología Molecular y Celular de Plantas. A collection of T-DNA activation tagged *Arabidopsis* lines in the ecotype C24 background was generated by utilizing the vector pSKI105, kindly provided by Detlef Weigel, Max Planck Institute for Developmental Biology.

### Production of Oligogalacturonides

*Escherichia coli* clones overexpressing the *Pectobacterium wasabiae* polygalacturonase gene *pehA* were generously provided by H. T. Saarilahti, University of Helsinki. The PehA protein was extracted and used to degrade commercially available polygalacturonic acid (Sigma–Aldrich) (Saarilahti et al., 1990). The concentration of the resulting OG-mixture was estimated in comparison to the concentrations of commercially available trimers on aluminum Silica gel 60_F254_ TLC-plates and analyzed using mass spectrometry to confirm that the preparation consisted of OGs with a DP between 2 and 19 (Supplementary Figure S5).

### Growth Inhibition Assay

*Arabidopsis thaliana* seeds were placed in individual wells of 12-well plates, with each well containing 1 ml of liquid ½12 MS medium (Gómez-Gómez et al., 1999). The plates were sealed with Parafilm to minimize evaporation. The seeds were stratified for 3 days at 4°C and then allowed to grow in a 12 h light cycle at 20°C. After 8 days, the medium was replaced with fresh liquid, and the seedlings were grown for 2 more days before treatment with OG-containing medium. After treatment, the plants were grown for 8 days before total plant weight was assessed. For all subsequent assays, four plants were combined into each biological replicate, and at least four biological replicates were tested per treatment. All experiments were performed at least three times. The average plant weight per treatment was compared across the treatments and statistically analyzed using Student’s *t-*test.

### Cuticular Permeability and Cell Wall Fortification Assays

Toluidine blue (TB) tests were performed to assess cuticle permeability as previously described by L’Haridon et al. (2011) with the following modifications. Briefly, 6 μL of TB solution (0.05%) in ½14 PDB were applied onto fully expanded leaves, then covered with a transparent cover for 1 h. After 1 h leaves were washed with tap water and photographed. The photos were used to quantify the sizes of staining area in Fiji^1^ as described by Cui et al. (2016). For staining with Calcofluor white (Supplementary Figure S4) leaves were decolorized overnight in 96% ethanol, incubated in 0.07 M sodium phosphate buffer (pH 9) for 1 h and incubated for 30 min in 0.05% Calcofluor white in 0.07 M sodium phosphate buffer (pH 9). Then leaves were rinsed in sodium phosphate buffer to remove excess of Calcofluor white, viewed and photographed under UV light on an InGenius LHR system (Syngene). To test cell wall fortification, the supernatant of an overnight culture filtrate of *Pectobacterium carotovorum* SCC1 was diluted 1:1 with water, and 1 μl of this diluted solution was placed on the adaxial side of the leaf surface of 3-week-old soil plants. The extent of maceration was documented using photography after 6 and 24 h.

### RNA Extraction and Quantitative RT-PCR Analysis

Plant seedlings were grown *in vitro* as described by Ferrari et al. (2007), except that the growth medium base used in this study was 1/2MS and the photoperiod was 12 h. The concentrations that were used in this study were set primarily to induce significant growth retardation and so that the induction of defense genes could be observed. Briefly, the protocol for treating plants was similar to that used in previously described growth inhibition studies (Gómez-Gómez et al., 1999), except that 12 seeds were placed in each well for germination. The seedlings were treated with OG-mix (200 μM) or a mock mixture (1/2MS) for 3 h prior to RNA extraction. For each biological replicate, the plants were harvested from 3 wells, rinsed in MQ water and blotted dry before they were frozen in liquid nitrogen. The plant material was then crushed using metal beads in a shaker. RNA was extracted using a GeneJET Plant RNA Purification mini kit (Thermo Scientific) according to the manufacturer’s protocol. Sample quality was assessed using gel electrophoresis and measured using a NanoDrop (Thermo Scientific). A 2 μg sample of total RNA was treated with RNAse-free DNaseI (Thermo Scientific) and then used as the template for cDNA synthesis with a Maxima Reverse Transcriptase and Ribolock RNase Inhibitor (Thermo Scientific) according to the manufacturer’s protocol. Random hexamers and oligo dT primers were used for cDNA synthesis. For qPCR, HOT FIREPol EvaGreen qPCR Mix Plus (Solis Biodyne) was used in a reaction containing approximately 8 ng of cDNA in a total reaction volume of 10 μl. The reactions were performed on a BioRad CFX using the following amplification program: an initial activation cycle of 95°C for 15 min, 40 cycles at 95°C for 15 s, 60°C for 20 s and 72°C for 20 s, and a final melt curve analysis. The resulting qRT-PCR data were analyzed using geNorm software (Vandesompele et al., 2002). The housekeeping genes that were used as the reference genes for the qRT-PCR analysis were AT4G05320 (*UBQ10*), AT5G09810 (*ACT7*), AT5G12250 (*TUB6*), AT5G60390 (*EF1α*) and AT3G13920 (*eIF4A*). These genes were tested to determine their stability across cDNA samples using the geNorm *M*-value principle in Genex 6 software (MultiD Analyses AB, Gothenburg, Sweden). The two most stable reference genes (*EF1α* and *UBQ10*) had *M*-values < 0.3 across all samples and were therefore used for subsequent normalizations (Vandesompele et al., 2002; Beekman et al., 2011). All of the genes that were analyzed, along with their respective primers, are listed in Supplementary Table S1.

### RNA Sequencing

Total RNA was extracted from 3 biological replicates per treatment (OG-mix and mock) and purified to remove rRNA using an Epicenter Ribo-Zero rRNA removal kit (Plant leaf) according to the manufacturer’s instructions. The quality and the amount of the extracted RNA was assayed using a QuBit fluorometer (Life Technologies) and a NanoDrop before sequencing. Sequencing was performed on a SOLiD 5500XL platform. Each sample was tested as part of a pool and run in two separate lanes to generate approximately 9 million 75 bp-long single-end reads that were subsequently mapped to the genome using the SHRiMP platform (Rumble et al., 2009). A total of 33,597 gene sequences were used to align the reads from the sequencing data. Gene expression was considered to be significantly different at FDR values < 0.05 and a log_2_ fold-change of ≥0.5 or ≤-0.5, as calculated using the R package DESeq2 (Love et al., 2014). The raw RNA sequencing data can be found in the NCBI GEO repository under the accession number GSE69538.

### Infection Assays

*Pectobacterium carotovorum* ssp. *carotovorum* SCC1 was cultured overnight in liquid Luria-Bertani (LB) medium at 28°C. The bacteria were pelleted using centrifugation and washed twice with and then resuspended in 10 mM MgSO_4_. The amount of bacteria in the infection suspension was adjusted to 1 × 10^5^ cfu/ml. Infection was initiated by wounding the surfaces of 4-week-old plant leaves with a pipette tip syringe and then applying 5 μl of bacterial solution to the wound site. Three leaves were infected per plant, and these three leaves were combined to represent one biological sample. The plants were covered with plastic lids to keep the moisture level high.

*Pseudomonas syringae pv. tomato* DC3000 was grown in 5 ml liquid King’s broth (KB) medium overnight at 28°C, washed twice with 10 mM MgSO4, and then resuspended in 10 mM MgSO4 supplemented with 0.025% silwet. The amount of bacteria was adjusted to 1 × 10^7^ cfu/ml and sprayed onto 4-week-old plants. The plants were covered with plastic lids to keep the moisture level high. At the indicated time points, three leaves were obtained from each plant, and a 0.5 cm^2^ leaf disk was harvested at each site of infection. The samples from one plant were combined to form one biological sample. The number of viable bacteria in each biological sample was then determined by counting cfus.

*Botrytis cinerea* Pers.:Fr strain B.05.10 was cultured on potato carrot agar (PCA) plates. Spores were harvested in ½14 strength potato dextrose broth (PDB) medium and filtered through Miracloth (Calbiochem) to remove hyphae. Droplets of 5 μL spore suspension (2 × 10^5^ spores mL^-1^) were applied to the leaves of 4-week-old plants for quantification of lesion size (mm) after 72 h. Three fully expanded leaves per plant were infected. The plants were covered with plastic lids to keep the moisture level high and transferred to growth chamber 180 μmol m^-2^ s^-1^, 8 h light/16 h dark at 23°C/18°C (day/night).

### Plant Transformation and Vectors

The following binary T-DNA destination vectors were used; pMDC32 (Curtis and Grossniklaus, 2003) and pHGWFS7.0 (Karimi et al., 2002). A 1834-bp sequence corresponding to the *PER57* promoter was amplified from Col-0 genomic DNA using primers listed in Supplementary Table S1. *Agrobacterium tumefaciens* carrying pMP90 was used to transform *A. thaliana* via floral dipping (Clough and Bent, 1998). T_0_ seeds were selected on MS medium containing the appropriate antibiotic. Stable transgenic lines showing either an increased expression level by qPCR or reporter gene signal were used for detailed analysis in this study.

### Detection of ROS

DAB and NBT staining for hydrogen peroxide and superoxide, respectively, were performed as described in Piisilä et al. (2015). *In situ* detection of hydrogen peroxide was performed by vacuum infiltration with 0.1% DAB (Diaminobenzidine tetrahydrochloride, Sigma–Aldrich) in 10 mM MES [2-(*N*-morpholino) ethanesulfonic acid], pH 6.5, for 30 min. For superoxide staining detached leaves were first vacuum infiltrated with 10 mM K_2_PO_4_ buffer, pH 7.8, for 30 min and then the buffer was replaced with 0.1% NBT (Nitroblue tetrazolium, Sigma–Aldrich) in 10 mM K_2_PO_4_ buffer and the leaves were left under table lights for 30 min. After staining the leaves were cleaned by boiling in alcohol-lactophenol (2:1) for 5 min, and then rinsed twice with 50% ethanol and once with MQ water.

### Callose Staining

*In situ* detection of callose was performed as described in Daudi et al. (2012). Briefly, three fully expanded leaves from 24- to 28-day-old *A. thaliana* plants were syringe-infiltrated with ∼100 μL of a diluted microbial elicitor solution (1 μM flg22 or 5 mM OG) and covered with a transparent cover to maintain high humidity for 20 h. At least eight independent plants were used as biological replicates. After 20 h incubation leaves were harvested and placed in sterile 12-well plates. Then washed with acetic acid:ethanol (1:3) for 6 h to destain the chlorophyll from the leaves followed by ethanol (50% v/v) 1 h, ethanol (30% v/v) 1 h and finally MQ water for 2 h. Leaves were stained with a phosphate buffer (pH 7.5) containing 5 mg/mL aniline blue (Sigma) for 20 h in the dark, transferred to 50% glycerol (Sigma) and examined under fluorescence microscopy. For the calculation of callose deposits, the following Fiji^2^ tools were used: ‘Auto Threshold,’ ‘Make Binary,’ and ‘Analyze Particles.’

### Apoplastic Peroxidase Extraction and Peroxidase Activity Assay

The apoplastic proteins were extracted from leaves using methods from Bindschedler et al. (2006). Briefly, apoplastic proteins were extracted from leaves by grinding them in liquid nitrogen with mortar and pestle. Powder was suspended in 100 mM Tris-HCl buffer pH 7.5 containing 250 mM sucrose and protease inhibitors and centrifuged at 16 100 g for 5 min at 4°C. Pellet was resuspended in 100 mM Tris-HCl pH 7.5 1 M NaCl, 1 mM CaCl_2_, 1 mM MgCl_2_ and ionically bound proteins from cell walls were extracted by vortexing 30 min at 4°C. After centrifugation at 16 100 *g* for 5 min at 4°C, and the supernatant was used as the crude extract for peroxidase activity assay. Total protein in the crude extract was estimated by Bradford assay using a Bio-Rad protein assay dye reagent (Biorad).

Peroxidase activity from the crude extract was determined from the rate of oxidation of guaiacol to tetraguaiacol, by monitoring the increase of absorbance in 470 nm (Li et al., 2008). Briefly, 10 μl of the crude extract was added to 150 μl of 5 mM sodium phosphate buffer pH 6.0, in 96 wells microplate. To establish back ground absorbance, 20 μl of 1 % (v/v) guaiacol (Sigma–Aldrich) in sodium phosphate buffer pH 6.0 was added to the reaction mixtures in the microplate and the absorbance at 470 nm was measured after 5 min incubation in room temperature. The oxidation reaction was started by adding 20 μl of 3% hydrogen peroxidase (Sigma–Aldrich) in sodium phosphate buffer pH 6.0 and the increase in absorbance at 470 nm was recorded for 3 min using a plate reader (EnSpire 2300, Perkin Elmer). Linear regression was applied to the absorbance data and peroxidase activity was calculated as a slope of the regression line. The peroxidase activity was normalized using the total protein content in the crude extract.

### Treatment with ABA and DPI

The effect of ABA and diphenylene iodonium (DPI), an inhibitor of NADPH oxidase-dependent oxidative burst, on cuticle permeability, ROS formation and pathogen infections was determined after leaves were sprayed under humid conditions with 100 μM ABA (Sigma–Aldrich) on developmental days 7 and 14, alternatively 50 μM DPI (Sigma–Aldrich) on developmental day 20. The permeability of the cuticles, ROS formation and symptoms of infection were evaluated 7 days post-ABA treatment and 24 h post-DPI treatment.

## Results

### Isolation and Characterization of the *ohy1* Mutant Line

**FIGURE 1:**
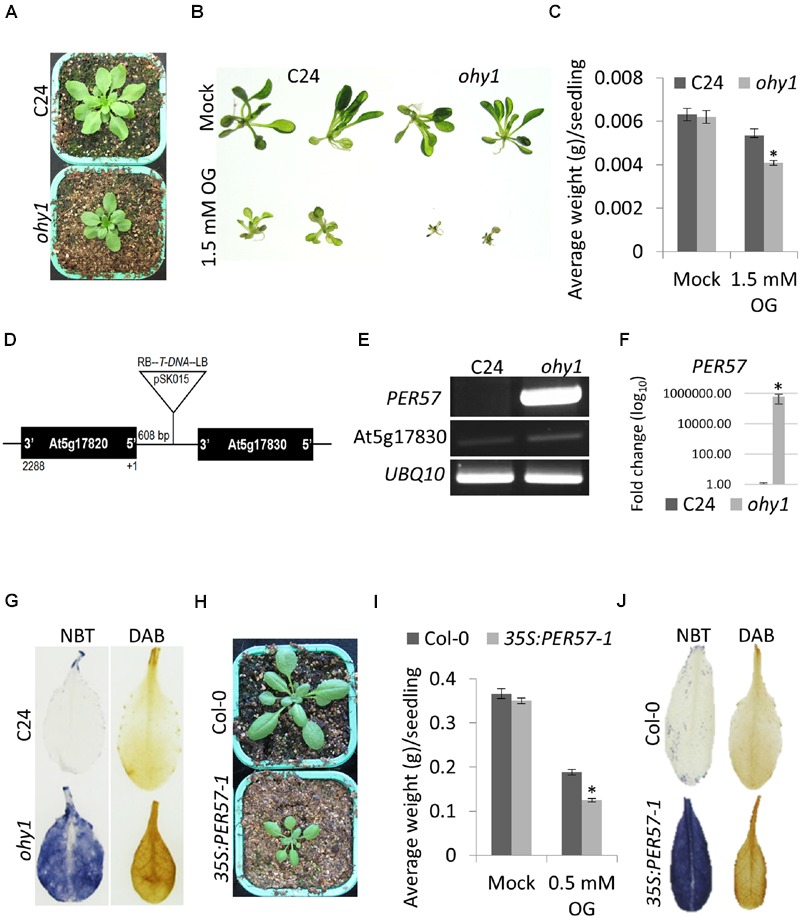
**Phenotypic and molecular characterization of *A. thaliana ohy1* plants. (A)** The growth phenotypes of 21-day-old C24 and *ohy1* plants. **(B)** The effect of 1.5 mM OGs on C24 and *ohy1* seedling growth. **(C)** Growth inhibition triggered by OGs in 10-day-old C24 and *ohy1* seedlings (*n* = 96, ±SD). ^∗^*p* < 0.05, Student’s *t-*test. **(D)** Location of the T-DNA insertion in *ohy1* plants. The T-DNA was inserted 608 bp upstream of the start codon of *PER57* (At5g17820), with the T-DNA right border oriented toward the ATG codon. **(E)** Gene expression of *PER57* and At5g17830 was measured from the leaves of 28-day-old C24 and *ohy1* soil-grown plants using RT-PCR. *UBQ10* was used as the internal control. **(F)** Gene expression of *PER57* was measured from the leaves of 28-day-old C24 and *ohy1* soil-grown plants using quantitative RT-PCR, normalized to *EF1α* and *UBQ10.* Error bars represent the standard deviation of three biological replicates. ^∗^*p* < 0.05, Student’s *t-*test. **(G)** The leaves of 28-day-old C24 and *ohy1* plants were stained using NBT and DAB to detect the accumulation of superoxide and hydrogen peroxide, respectively. **(H)** Growth phenotypes of 21-day-old Col-0 and *35S:PER57-1* plants. **(I)** Growth inhibition triggered by OGs in 21-day-old Col-0 and *35S:PER57-1* seedlings (*n* = 48, ± SD). ^∗^*p* < 0.05, Student’s *t-*test. **(J)** Leaves of 28-day-old Col-0 and *35S:PER57-1* plants were stained using NBT and DAB to detect the accumulation of superoxide and hydrogen peroxide, respectively. All experiments were repeated at least three times with similar results.

To identify and characterize OG-responsive components that are involved in innate immunity, a population of 62000 T-DNA activation tagged *A. thaliana* ecotype C24 lines, generated previously in our laboratory by using the vector pSKI015 (Weigel et al., 2000), were treated with OG elicitors. The screen was based on OG-triggered growth retardation of *A. thaliana* seedlings grown in liquid ½12 MS on 96-well plates that allowed high throughput detection of the mutant phenotypes. The screen yielded 46 activation-tagged mutant lines impaired in their growth responses to OGs. Out of the 46 activation-tagged mutant lines that displayed impaired OG responses, one mutant line that was called *ohy1* (OG hypersensitive 1) exhibited a strong hypersensitive response to OGs (DP 2–19) (**Figures 1B,C**) and was therefore chosen for further characterization. The *ohy1* mutant was smaller in size (**Figure 1A**) than the wild-type plants. A co-segregation analysis of progeny obtained from backcrosses of *ohy1* and wild-type plants indicated that the mutation linked to the observed phenotypes was dominant and that the phenotype was dependent on the inserted T-DNA. We used next-generation Illumina sequencing to track the location of the T-DNA insertion in the *ohy1* mutant within the extragenic DNA region between genes At5g17820 and At5g17830. The CaMV 35S enhancers that were present in the T-DNA were found to be 608 bp upstream of the At5g17820 (*PER57*) start codon (**Figure 1D**), and the expression of the *PER57* gene encoding a CIII peroxidase was clearly enhanced (**Figures 1E,F**).

The observed overexpression of *PER57* in *ohy1* suggested that the accumulation of ROS might be enhanced in these plants. To test this, we characterized hydrogen peroxide and superoxide levels in *ohy1* and wild-type plants using diaminobenzidine (DAB) and nitroblue tetrazolium (NBT) staining, respectively (**Figure 1G**). Indeed, the overexpression of *PER57* resulted in higher levels of superoxide and hydrogen peroxide accumulation in the leaves of the *ohy1* plants than in the wild-type plants, indicating enhanced peroxidase activity (**Figure 1G**). To confirm that the up-regulation of *PER57* in *ohy1* plants was responsible for the observed phenotypes, we used the CaMV35S promoter to drive the expression of the *PER57* cDNA in wild-type Col-0 plants. Of the three independent transgenic lines that were analyzed, all lines displayed phenotypes that were identical to the *ohy1* mutant in that they were smaller than wild-type plants (**Figure 1H**) and exhibited both hypersensitivity to OG elicitors (**Figure 1I**) and an increase in the accumulation of superoxide and hydrogen peroxide (**Figure 1J**). The phenotypes observed in the gain-of-function transgenic lines confirmed that the increased expression of *PER57* was indeed responsible for the phenotypes that were observed in the *ohy1* mutant plants.

### Overexpression of *PER57* Primes OG Signaling and Confers Resistance to Necrotrophic Pathogens and Sensitivity to Hemibiotrophic Pathogens

**FIGURE 2:**
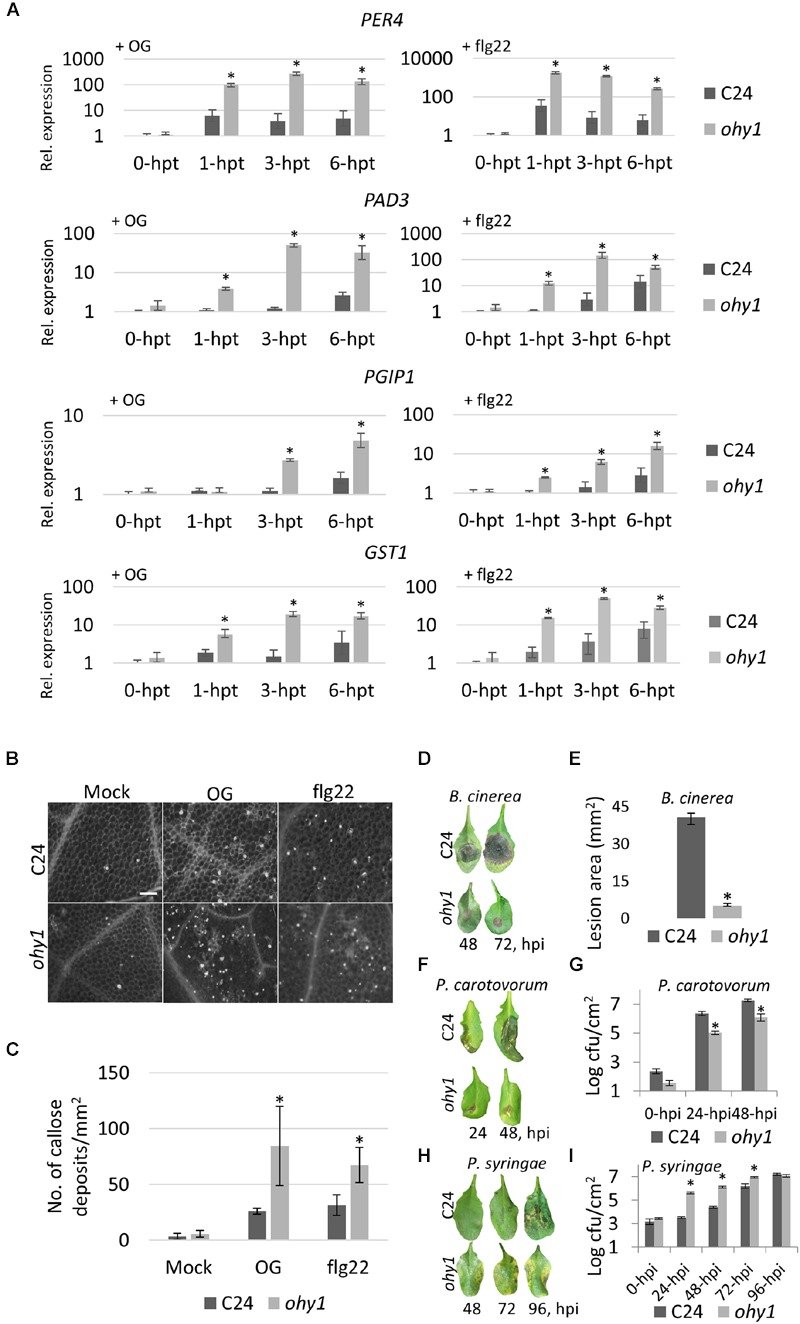
**Overexpressing *PER57* primes the expression of OG-responsive defense-related genes and confers resistance to necrotrophic bacterial and fungal pathogens. (A)** Defense gene induction in response to 200 μM OGs or 100 nM flg22 of C24 and *ohy1* seedlings. Gene expression levels of the OG-induced *PAD3, PGIP1*, and *PER4* marker genes and wound-induced *GST1* marker gene were measured using quantitative RT-PCR analysis, normalized to *EF1α* and *UBQ10* (reference genes) and plotted relative to C24 at the 0-hpt expression level. Total RNA was extracted from the leaves of 28-day-old *ohy1* plants that were grown on soil. Error bars represent the standard deviation of three biological replicates. ^∗^*p* < 0.05, Student’s *t-*test. **(B)** Leaves on 28-day-old C24 and *ohy1* plants were infiltrated with 5 mM OGs and 1 μM flg22 for 24 h and then stained with aniline blue to visualize callose. **(C)** Quantification of OG- and flg22-induced callose deposits acquired from leaves of 28-day-old C24 and *ohy1* plants. Number of callose deposits per 1 mm^2^ were counted by Fiji using functions ”Auto Threshold,” ”Make Binary” and ”Analyze Particles” (*n* = 8, ±SD). ^∗^*p* < 0.05, Student’s *t-*test. Scale bar, 200 μm. **(D)** Five-microliter droplets of a suspension of *B. cinerea* spores (2 × 10^5^ spores mL^-1^) were placed on the leaves of 28-day-old C24 amd *ohy1* plants. Disease symptoms were analyzed at 48 and 72 h post-infection (hpi). **(E)** Images were taken 3 days post inoculation with *B. cinerea*. The lesion area of 28-day-old C24 and *ohy1* plants was measured in Fiji (*n* = 8, ±SD). ^∗^*p* < 0.05, Student’s *t-*test. **(F)** Disease symptoms were evaluated at 24 and 48 hpi with *P. carotovorum* in 28-day-old C24 and *ohy1* plants. **(G)** Growth of *P. carotovorum* was evaluated at 24 and 48 hpi in 28-day-old C24 and *ohy1* plants (*n* = 5, ±SD). ^∗^*p* < 0.05, Student’s *t-*test. **(H)** Disease symptoms were evaluated at 48, 72, and 96 hpi with *P. syringae* in 28-day-old C24 and *ohy1* plants. **(I)** Growth of *P. syringae* was evaluated at 24, 48, 72, and 96 hpi in 28-day-old C24 and *ohy1* plants (*n* = 3, ±SD). ^∗^*p* < 0.05, Student’s *t-*test. All experiments were repeated at least twice with similar results.

Based on the finding that the overexpression of *PER57* conferred enhanced sensitivity to OGs, manifested as increased growth retardation and ROS formation (**Figure 1**), we hypothesized that *ohy1* plants might also exhibit enhanced DAMP-responses, including an increase in the expression of OG-marker genes. To explore this hypothesis, we treated plants with OGs and monitored the expression of known markers of the OG (*PAD3, PER4, PGIP1* and *GST1*), SA (*PR1, PR5*, and *SID2*) and JA (*VSP2*) signaling pathways. The *ohy1* line showed no significant changes in markers of SA and JA signaling in response to OGs (Supplementary Figure S1). In contrast, the expression levels of the OG signaling marker genes were much higher in *ohy1* plants than those observed in the wild-type controls (**Figure 2A**). Interestingly, the expression levels of defense genes in non-treated *ohy1* plants were similar to those in wild-type plants, but faster and stronger induction was observed in response to OGs in the *ohy1* plants. Consequently, we hypothesized that defense responses in general could be primed in the *ohy1* mutant plants. To confirm this, we analyzed the level of induction of defense genes in response to the bacterial PAMP flg22 (**Figure 2A**) and the accumulation of callose in response to both OG- and flg22 elicitors (**Figure 2B**). We found that flg22 triggered a stronger level of expression of defense genes in *ohy1* than those observed in the wild-type controls. In agreement with the observation of an enhanced expression of defense genes in *ohy1*, we also observed an increase in callose deposits in response to both elicitors (**Figures 2B,C**), which support the notion that defense responses are indeed primed in *ohy1* plants.

Consistent with the stronger induction of defense-related genes and enhanced callose deposition in the *ohy1* line, we hypothesized that the response to pathogens could also be enhanced in these plants. To explore the potential effect of the enhanced expression of *PER57* in disease resistance, we infected *ohy1* and wild-type plants with two necrotrophic pathogens: the soft-rot bacterium *P. carotovorum* and the gray mold fungus *B. cinerea*. Interestingly, the *ohy1* mutant displayed immunity to *B. cinerea* and a clearly higher level of resistance to *P. carotovorum* in comparison to the wild-type controls (**Figures 2D–G**). To determine whether *ohy1* plants demonstrated enhanced resistance to other than necrotrophic pathogens, we infected mutant and wild-type plants with the hemibiotroph *P. syringae* (**Figures 2H,I**). Surprisingly, in contrast to the observed increase in resistance to necrotrophic pathogens, the *ohy1* line was clearly more susceptible than the wild-type plants to *P. syringae*. These results indicate that the overexpression of *PER57* in the *ohy1* line primes plant defenses to necrotrophic pathogens, while sensitizing the plant to pathogens with a hemibiotrophic lifestyle.

### Overexpression of *PER57* Increases Cuticle Permeability by Modulating the Expression of Cutin- and Wax-Biosynthetic Genes

**FIGURE 3:**
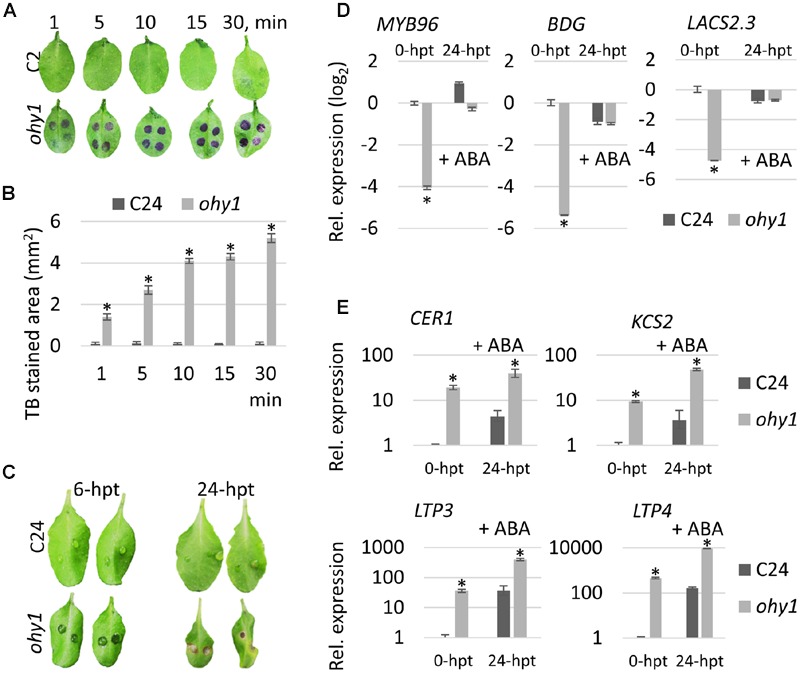
**Overexpression of *PER57* increases the permeability of the leaf cuticle. (A)** Toluidine blue (TB) exclusion assay. Leaves of 21-day-old C24 and *ohy1* plants were stained with 6 μl of 0.05 % TB drops at indicated time points (*n* = 8). **(B)** TB-stained areas were quantified in Fiji (*n* = 8, ±SD). ^∗^*p* < 0.05, Student’s *t-*test. **(C)** C24 and *ohy1* plants were treated with CWDEs. CWDEs, when applied to plant tissue, cause symptoms that can include tissue maceration and cell death. Cell wall fortification was assessed 6 and 24 h post-treatment (hpt) with CWDEs (*n* = 6). **(D)** Quantitative RT-PCR analysis was performed to evaluate the levels of *MYB96, BDG* and *LACS2.3* transcripts in 28-day-old *ohy1* rosette leaves at 24 h after the plants were sprayed with a mock solution or 100 μM ABA. Transcript levels were plotted relative to the expression level in the C24 line at 0-hpt. The *EF1α* and *UBQ10* reference genes were used as internal controls. Error bars represent the standard deviation of three biological replicates. ^∗^*p* < 0.05, Student’s *t-*test. **(E)** Quantitative RT-PCR analysis was performed to evaluate the levels of *CER1, LTP3, LTP4* and *KCS2* transcripts in 28-day-old *ohy1* rosette leaves at 24 h after the plants were sprayed with a mock solution or 100 μM ABA. Transcript levels were plotted relative to the levels in the C24 line at 0-hpt. The *EF1α* and *UBQ10* reference genes were used as internal controls. Error bars represent the standard deviation of three biological replicates. ^∗^*p* < 0.05, Student’s *t-*test. All experiments were repeated twice with similar results.

Our results (**Figure 2**) showed that the overexpression of *PER57* in the *ohy1* plants conferred resistance to *B. cinerea* and *P. carotovorum.* Interestingly, similar resistance phenotypes have been observed in plants with cuticular defects showing immunity to *B. cinerea* (Bessire et al., 2007; Voisin et al., 2009; Chassot et al., 2007; L’Haridon et al., 2011). This similarity prompted us to explore whether the permeability of the leaf cuticle was increased in the *ohy1* mutant. We examined the permeability of the cuticle of *ohy1* leaves by applying the hydrophilic agent TB (Tanaka et al., 2004) to the adaxial sides of the leaves (**Figures 3A,B**). As expected, no staining was observed in the leaves of wild-type plants, but dark blue staining was clearly visible in the leaves of *ohy1* mutant plants as early as 1 min after the TB was applied. These results indicate that the permeability of the leaf cuticle was increased in the *ohy1* plants (**Figures 3A,B**). This increase in cuticular permeability could be associated with a decrease in the fortification of the cell walls. When drops of the *P. carotovorum* cellular culture filtrates enriched in cell wall-degrading enzymes (CWDE) were applied to the adaxial side of the leaves, increased leaf maceration was observed in *ohy1* plants in comparison to the wild-type controls (**Figure 3C**). This data indicates a clear reduction in the fortification of the cell walls of the *ohy1* plants.

To explore the mechanisms by which peroxidase-generated ROS increase permeability in leaf cuticles, we first hypothesized that these ROS might have a negative effect on the expression of genes that affect cutin biosynthesis. Therefore, the expression levels of *MYB96*, a positive regulator of cutin formation (Seo et al., 2011; Cui et al., 2016), and *BDG* and *LACS2.3*, which are both major cutin-biosynthetic genes, were analyzed in the *ohy1* line. The expression levels of these genes were strongly down-regulated in the *ohy1* line, suggesting that the loss of cuticle integrity was influenced by impaired cutin biosynthesis (**Figure 3D**). In addition, the enhanced accumulation of cuticular waxes has been observed in *bdg, lacs2.3, fdh* and *lcr* mutants that display impaired cuticle integrity and may suggest a mechanism involving a compensatory reaction to the loss of cuticle integrity (Kurdyukov et al., 2006; Bessire et al., 2007; Voisin et al., 2009). To test whether this was the case also with *ohy1* plants, we characterized the expression of the cuticular wax-biosynthetic genes *CER1, LTP3, LTP4*, and *KCS2* in wild-type and *ohy1* plants. As expected, the expression levels of these genes were much higher in the *ohy1* plants than in the wild-type controls (**Figure 3E**).

In summary, our results indicate that CIII peroxidase-derived ROS in the *ohy1* plants modulate the expression of genes known to be associated with cuticle biosynthesis, which leads to permeabilization of the cuticle.

### The *ohy1* Can be Phenocopied by Other CIII Peroxidases

**FIGURE 4:**
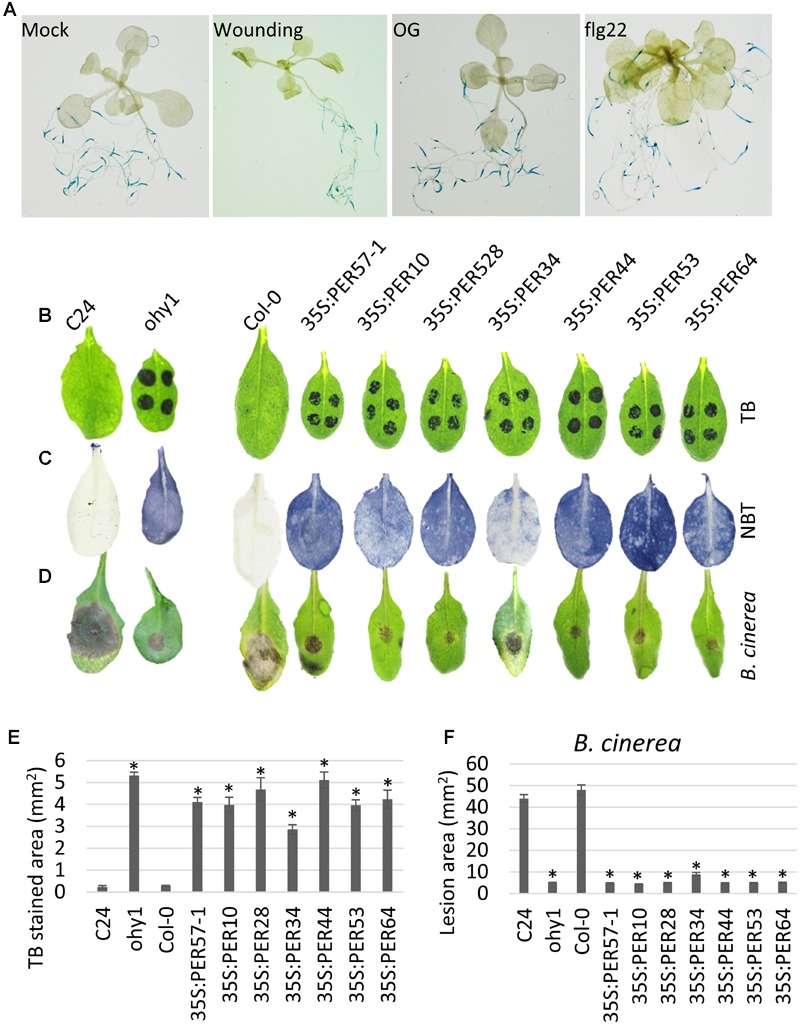
**Overexpression of different CIII peroxidases phenocopies the overexpression of *PER57.* (A)** The expression of *pPER57::GUS* was evaluated in 21-day-old Col-0 seedlings sprayed with 5 mM OGs or 1 μM flg22 24-hpt, alternatively the leaves were wounded by gently pressing the lamina with laboratory forceps. **(B)** Toluidine blue (TB) staining was used to indicate cuticle permeability in 24-day-old C24, *ohy1*, Col-0 and transgenic lines overexpressing the apoplastic peroxidases *PER57, PER10, PER28, PER34, PER44, PER53*, and *PER64* (*n* = 4). **(C)** Superoxide formation was detected using NBT staining in 21-day-old C24, *ohy1*, Col-0 plants and plants that overexpressed apoplastic peroxidases (*n* = 4). **(D)** Disease symptoms were evaluated at 3 days post-infection (dpi) with *B. cinerea* in 28-day-old C24, *ohy1*, Col-0 plants and plants overexpressing apoplastic peroxidases (*n* = 3). **(E)** TB-stained areas were quantified in Fiji. (*n* = 4, ±SD, *N* = 12 in total from three independent experiments). ^∗^*p* < 0.05, Student’s *t-*test. **(F)** Five-microliter droplets of a suspension of *B. cinerea* spores (2 × 10^5^ spores mL^-1^) were placed on the leaves of 28-day-old plants. Images were taken three days post inoculation. The lesion area was measured in Fiji (*n* = 8, ± SD). ^∗^*p* < 0.05, Student’s *t-*test. All experiments were repeated at least twice.

To determine the expression profile of *PER57 in vivo*, we generated transgenic *A. thaliana* plants that expressed the GUS reporter gene under the control of the *PER57* promoter. The generated transgenic plants were sprayed either with OGs or flg22, or wounded with laboratory forceps. GUS-staining of the transgenic plants indicated that *PER57* appears to be specifically expressed in roots and no GUS-activity was detected in leaves even in response to wounding or flg22 and OG elicitors (**Figure 4A**).

CIII peroxidases exist as large multigene families and often appear to have redundant functions (Cosio and Dunand, 2009), several of which are expressed in leaves, which could explain the effect obtained by ectopic expression of *PER57*. Consequently, overexpressing other CIII peroxidase genes might result in the same phenotypes that were observed in the *PER57* overexpressors, including the *ohy1* mutant. To test if the phenotypes observed in *PER57* overexpressors is a general effect of elevated levels of CIII peroxidases, we generated transgenic lines that overexpressed six different CIII peroxidases, which, according to our RNA sequence analysis of 10-day-old OG-treated *A. thaliana* seedlings (Supplementary Table S2; Supplementary Figure S3) were either responsive: *PER10, PER28*, and *PER34*, or non-responsive: *PER44, PER53*, and *PER64* to OG elicitors. Overexpression of all six CIII peroxidase genes resulted in phenotypes that were identical to the phenotype of the *ohy1* mutant, including an increase in the permeability of the leaf cuticle (**Figures 4B,E**), a dramatic increase in the formation of superoxide (**Figure 4C**) and a strong increase in resistance to *B. cinerea* (**Figures 4D,F**). The results demonstrated that the effect of *PER57* overexpression on cuticular integrity and resistance to pathogens is not specific and the same effect could be achieved by overexpressing other apoplastic CIII peroxidases.

### Peroxidase Activity Is Elevated in ABA Biosynthesis- and Response Mutants

Abscisic acid has been shown to influence cuticle formation in several studies (Asselbergh et al., 2007; L’Haridon et al., 2011). To test whether this was the case in CIII peroxidase overexpressors, we analyzed the expression levels of *MYB96, BDG* and *LACS2.3* in *ohy1* plants that were treated with ABA. Interestingly, applying ABA completely restored normal expression levels of these genes in the *ohy1* line (**Figure 3D**). Furthermore, similar to the effect of ABA on the expression of cutin biosynthesis genes, the transcription of *CER1, LTP3, LTP4*, and *KCS2* was increased after ABA treatment (**Figure 3E**). Apparently, ABA plays an important role in activating genes known to be involved in cuticular wax biosynthesis.

**FIGURE 5:**
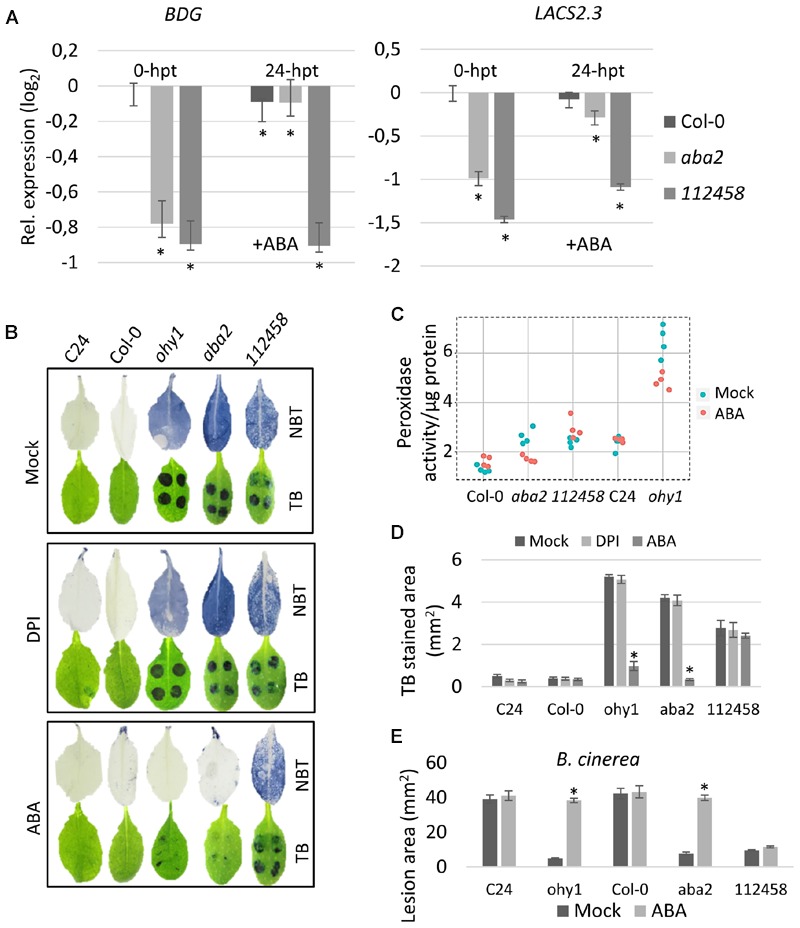
**Extensive ROS accumulation and increased peroxidase activity suppress the expression of cutin-biosynthetic genes in ABA mutants. (A)** Quantitative RT-PCR was performed to analyze the levels of *BDG* and *LACS2.3* transcripts in rosette leaves of 28 day-old *aba2* and *pyr/pyl 112458* mutants at 24 h after the plants were sprayed with a mock solution or 100 μM ABA. Transcript levels were plotted relative to the expression level in the C24 line at 0-hpt. The *EF1α* and *UBQ10* reference genes were used as internal controls. Error bars represent the standard deviation of three biological replicates; *t*-test ^∗^*p* < 0.05. **(B)** Phenotypes of 21-day-old C24, Col-0, *ohy1, aba2-1*, and *pyr/pyl 112458* leaves that were sprayed with 100 μM ABA on developmental days 7 and 14 or with 50 μM DPI on day 20. Superoxide formation was detected using NBT in 21-day-old plants. Cuticle permeability was assessed using TB in 21-day-old plants. **(C)** The peroxidase activity in ionically bound protein fractions was determined in 28-day-old C24, Col-0, *ohy1, aba2-1*, and *pyr/pyl 112458* plants (*n* = 4, ±SD). **(D)** TB-stained areas were quantified in Fiji (*n* = 4, ±SD, *N* = 8 in total from two independent experiments). ^∗^*p* < 0.05, Student’s *t-*test. **(E)** C24, Col-0, *ohy1, aba2-1*, and *pyr/pyl 112458* plants were sprayed with a mock solution or 100 μM ABA on developmental days 7 and 14. The plants were subsequently infected with *B. cinerea* when they were 21 days old. The lesion area was assessed at 3 days post-inoculation (*n* = 8, ±SD). ^∗^*p* < 0.05, Student’s *t-*test. All experiments were repeated at least twice with similar results.

Our data suggest that both CIII peroxidase-derived ROS and perturbed ABA levels affected the expression of genes known to be associated with cuticle biosynthesis. To further study the effect of ABA-signaling in cuticle permeability, we next sought to determine the expression levels of *BDG* and *LACS2.3* in ABA-deficient *aba2* and ABA-insensitive *pyr/pyl 112458* sextuple (Gonzalez-Guzman et al., 2012) mutant plants treated either with mock or ABA. The cuticular genes *BDG* and *LACS2.3* were strongly down-regulated in the mock-treated *aba2* and *pyr/pyl 112458* mutant plants (**Figure 5A**). Exposure to ABA restored the expression of these cuticular genes in the *aba2* mutant. However, as predicted, the expression of these genes remained strongly down-regulated in the ABA-insensitive *pyr/pyl 112458* mutant (**Figure 5A**).

Because we observed a strong positive correlation between higher levels of peroxidase-derived ROS and increased cuticle permeability (**Figures 5B,D**), we next sought to explore the effect of ABA on ROS formation in the *ohy1* mutant, ABA biosynthesis mutant *aba2*, and ABA signaling mutant *pyr/pyl 112458*. The mutant and wild-type plants were sprayed with 100 μM ABA, which resulted in the removal of ROS in all the lines except for the ABA insensitive *pyr/pyl 112458* plants. Furthermore, the ABA-induced decrease in the accumulation of ROS was accompanied by the restoration of the leaf cuticle (**Figure 5B**).

To determine whether NADPH oxidase contributed to the accumulation of ROS in the plants with increased leaf cuticle permeability, we sprayed these plants with 50 μM DPI, which is an inhibitor of NADPH oxidase-dependent oxidative bursts (**Figure 5B**). However, DPI had no significant effect on ROS formation nor on cuticle permeability (**Figure 5D**) in any of the tested lines, indicating that these ROS are likely to be produced primarily by peroxidases and not by NADPH oxidases.

We further performed peroxidase activity assays to determine whether ABA had an effect on peroxidase activities in *ohy1, aba2* and *pyr/pyl 112458* mutant plants. The plants were sprayed either with ABA or mock (**Figure 5C**). In the absence of ABA, all of the tested mutant plants exhibited higher levels of peroxidase activity than were observed in the wild-type plants. Interestingly, the addition of ABA reduced peroxidase activity to wild-type levels only in *aba2-1* mutant. While no significant changes were observed in peroxidase activity in the ABA-insensitive *pyr/pyl 112458* mutants, the *ohy1* mutant plants continued to demonstrate relatively high peroxidase activity despite their exposure to ABA. These data suggest that while the application of ABA outpaces the constitutive production of ROS by the peroxidases, the ABA-induced removal of ROS has little effect on the actual activity of the peroxidases.

Based on the finding that the application of ABA had a significant impact on the integrity of the cuticle, we suspected that ABA might also influence the enhanced pathogen resistance that was observed in the *ohy1* line and in the mutants with impaired ability to respond to or biosynthesize ABA. Indeed, pre-treatment of the *ohy1* and *aba* mutants with 100 μM ABA, led to wild-type level susceptibility to *B. cinerea*, whereas no alterations in the susceptibility was detected in the ABA-insensitive *pyr/pyl 112458* sextuple mutant (**Figure 5E**).

In summary, our results show a negative effect of ABA on ROS formation, and point to the involvement of CIII peroxidases as a source of ROS in *aba* mutants.

## Discussion

Here, we characterized an activation tagged *A. thaliana* line that overexpresses the CIII peroxidase *PER57*. This transgenic line, referred to as *ohy1*, is hypersensitive to OG elicitors and exhibited enhanced accumulation of ROS associated with increased cuticle permeability, and clearly enhanced resistance to necrotrophic fungal *B. cinerea* and bacterial *P. carotovorum* pathogens. The *ohy1* plants also exhibited increase in the OG- and flg22-triggered accumulation of callose, and enhanced expression of OG-responsive genes involved in plant defense responses. The overexpression of *PER57* in Col-0 background resulted in similar responses as those observed in the *ohy1* mutants, confirming the contribution of this peroxidase to the phenotype of the *ohy1* plants (**Figure 1**). However, the analysis of transgenic plants harboring GUS-fusions to *PER57* promoter (**Figure 4A**) demonstrated that the expression of the *PER57* is restricted to roots suggesting that the phenotypes seen in *ohy1* might not be specific for ectopic expression of *PER57* but could be obtained by expressing other CIII peroxidases. This indeed was the case as overexpression of six different CIII peroxidases, including peroxidases that are either responsive or non-responsive to OGs conferred a phenotype that was identical to that observed in the *ohy1* plants (**Figure 4B**). This indicates a more general involvement of CIII peroxidases in plant defense responses, an assumption that is supported by our results of RNA sequencing performed from OG-treated *A. thaliana* seedlings. In this analysis 19 CIII peroxidases were found to be up-regulated in response to these elicitors (Supplementary Table S2). In addition, it is worth noting that the contribution of PER57 appears to be insignificant to basal resistance to pathogens because *per57-1* plants display a wild-type level of susceptibility (Supplementary Figure S2). These results indicated that the increase in cuticular permeability resulting from enhanced activity of CIII peroxidases appears to be a general phenomenon and not dependent on any specific peroxidase. Furthermore, it did not seem to matter whether or not the expression of a CIII peroxidase was up-regulated in *A. thaliana* in response to defense elicitors. The overexpression of any CIII peroxidase, whether it was responsive or non-responsive, such as *PER57*, resulted in an identical phenotype. Therefore, we concluded that we can use the *ohy1* plants as a model to characterize the role of CIII peroxidases in *A. thaliana*.

How does an overexpression of *PER57* lead to a strong resistance to *B. cinerea* and *P. carotovorum* in *ohy1* plants? Our data indicate that the transgenic plants examined in this study are primed to initiate a subset of defense responses. For example, treatment with either of two elicitors (OGs or flg22) triggered a clearly stronger and faster expression of OG-responsive genes involved in plant defense in *ohy1* plants than in wild-type plants (**Figure 2A**). One could speculate that the primed defense response in *ohy1* plants might simply reflect an increase in the amount of elicitor that is capable of diffusing across the more permeable cuticle. Therefore, we further analyzed priming in leaves that were infiltrated with OG and flg22, bypassing the cuticle. For these experiments, we assessed callose deposition, which has emerged as a popular model system that can be used to quantify the activity of a plant’s immune system. Infiltrated *ohy1* plants continued to demonstrate an increase in callose deposition compared to wild-type, which strongly indicated that the phenotype of these plants resulted from primed defenses (**Figures 2B,C**). Furthermore, we show that the overexpression of *PER57* did not affect the expression of SA- and JA-responsive target genes (Supplementary Figure S1). The significant role of the OG signaling pathway in resistance to necrotrophic pathogens is in line with what has been reported in earlier research (Ferrari et al., 2007; reviewed in Davidsson et al., 2013). Enhanced resistance to *B. cinerea* was observed in CUTE plants in the absence of elicitation and independently of SA, ET and JA signaling (Chassot et al., 2007). Moreover, pretreating wild-type plants with OGs triggered the up-regulation of the OG-responsive antifungal compounds encoded by *PAD3* and *PGIP*, and improved resistance to *B. cinerea*. Accordingly, when a *pad3* mutant was treated with OGs, it did not display enhanced resistance to *B. cinerea* (Ferrari et al., 2007). These observations clearly suggest that the OG signaling pathway plays a key role in defenses against necrotrophic fungal pathogens.

We also demonstrated that overexpressing *PER57* led to an enhanced accumulation of transcripts of the lipid transfer proteins LTP3/4 (**Figure 3E**). These findings are supported by a study by Chassot et al. (2007) that was conducted using CUTE (cutinase-expressing) plants, which are characterized by a partly absent cuticle and nearly complete immunity to *B. cinerea*. In CUTE plants, the expression of members of three gene families that encode lipid transfer proteins, proteinase inhibitors and importantly, peroxidases were highly up-regulated in response to *B. cinerea* (Chassot et al., 2007). Overexpressing selected peroxidases in *A. thaliana* resulted in an increase in resistance to *B. cinerea*, and these results further support the notion that class III peroxidases are involved in defensive responses against necrotrophic fungal pathogens (Chassot et al., 2007). In addition, the peroxidases PER33 and PER34 have been shown to play major roles in ROS production in response to fungal and bacterial pathogens in *A. thaliana* (Daudi et al., 2012; O’Brien et al., 2012). The *per33*/*per34* knockdown plants exhibited impaired ability to produce callose depositions and lower expression levels of defense-related genes in response to a fungal elicitor. Unexpectedly, *rbohD* mutant was also compromised in callose deposition following elicitation with OGs. However, neither OG-elicited defense gene induction nor the protective effect of OGs against subsequent fungal infection was affected in the *rbohD* mutant (Galletti et al., 2008; Daudi et al., 2012; O’Brien et al., 2012). These results suggest that peroxidase-generated ROS might be a major component in PTI.

In previous studies, strong resistance to *B. cinerea* was also observed in wounded plants and in plants with cuticular defects (Chassot et al., 2008; L’Haridon et al., 2011). In both cases, ROS production levels were strongly correlated with enhanced resistance to *B. cinerea* (L’Haridon et al., 2011). Moreover, the *lacs2* mutant, which is defective in cutin biosynthesis, was found to be more susceptible to *P. syringae* and more resistant to *B. cinerea* (Tang et al., 2007). These phenotypes strongly resemble those we observed in CIII peroxidase overexpressors, which promoted us to study the mechanism behind the increased permeability.

**FIGURE 6:**
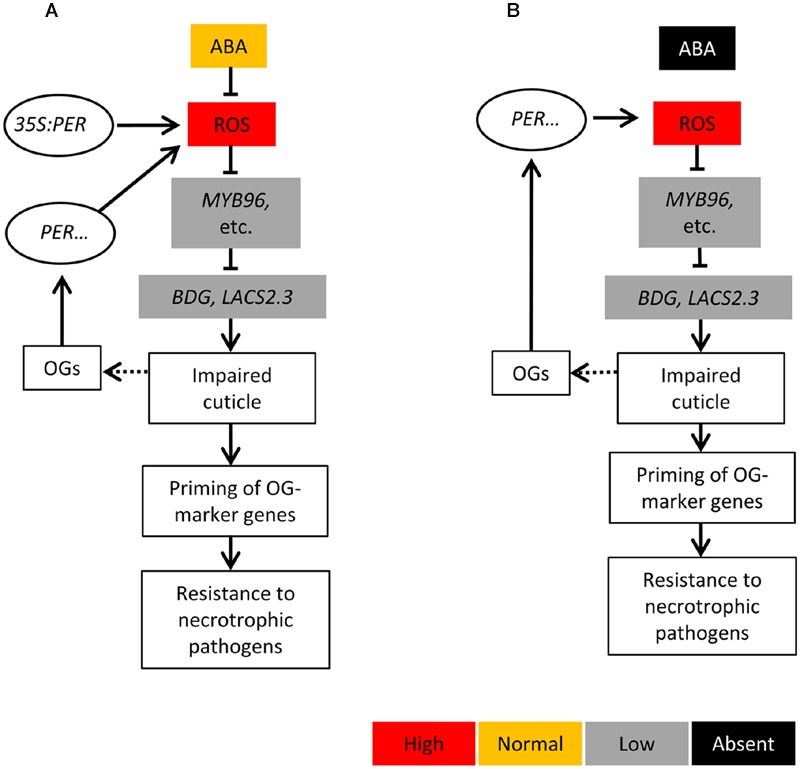
**Schematic model illustrating the conclusions.** The role of CIII peroxidase-generated ROS in cuticle formation. **(A)** The constitutively enhanced accumulation of peroxidase-generated ROS was positively correlated with the increased permeability of the cuticle and negatively correlated with ABA-mediated signaling. Cuticular integrity was influenced by the repression of genes involved in cutin biosynthesis. Under conditions involving a perturbed cuticle, plants switch to a primed state that includes enhanced OG-mediated defenses. **(B)** In ABA-deficient plants (*aba2-1*), the ABA-mediated removal of ROS is lost. This allows for an enhanced accumulation of peroxidase- and probably other enzyme-derived ROS, which subsequently represses the expression of cutin-biosynthetic genes and thereby leads to an increase in the permeability of the cuticle. This increased permeability is positively correlated with defense priming and induced resistance.

Here, we demonstrated that the enhanced expression of *PER57* in *ohy1* plants strongly reduced the expression of *MYB96*, a positive regulator of cuticle formation, in addition to *BDG* and *LACS2.3*, two major genes known to be involved in cuticle biosynthesis. Our data is supported by previous studies using the *bdg* and *lacs2.3* cuticular mutants (Schnurr et al., 2004; Bessire et al., 2007; L’Haridon et al., 2011; Jakobson et al., 2016), in which the permeability of the cuticle, the accumulation of ROS and cuticular waxes and the resistance to *B. cinerea* were enhanced in comparison to that observed in wild-type plants. The above findings, when taken together, indicate that in plants, cuticle integrity is influenced by a feedback mechanism. The impaired expression of cutin-biosynthetic genes in cuticular mutants was associated with the enhanced production of ROS, whereas the enhanced production of peroxidase-derived ROS was associated with a decrease in gene expression that led to more permeable cuticle (**Figures 3D** and **6**). Interestingly, ABA appears to influence the feedback loop between ROS and cuticle formation. First, a strong accumulation of ROS and the repression of the cuticle formation-related genes *BDG* and *LACS2.3* were observed in the ABA-deficient *aba2* mutant (**Figure 5A**). ABA-deficient *aba2* and *aba3* mutants have been reported to exhibit elevated levels of ROS and increased cuticle permeability, whereas applying ABA to these mutants was found to decrease cuticle permeability (L’Haridon et al., 2011; Cui et al., 2016). The accumulation of ROS in *ohy1* and *aba* mutant plants appears to originate from peroxidases, as application of the NADPH oxidase inhibitor DPI did not prevent the formation of ROS in tested mutant plants (**Figure 5B**). Further support of our data is provided by the previous observation that the accumulation of ROS was induced in the *NADPH oxidase D* (*rbohD*) and *F* (*rbohF*) mutants in addition to the double mutant *rbohD*/*F* in response to wounding (L’Haridon et al., 2011). Second, exogenously applied ABA inhibited ROS formation in both the *aba2* and the *ohy1* mutant (**Figure 5B**). These results are in line with previous studies that reported a negative role for ABA in ROS formation (Asselbergh et al., 2007; L’Haridon et al., 2011). Our observation that ROS were completely removed by exogenous ABA from *ohy1* plants, in which high levels of ROS are constitutively generated, suggests that the ABA-triggered removal of ROS is very efficient, even overcoming the constitutive production by CIII peroxidases. This notion is further supported by the observation that despite the efficient removal of ROS by ABA, the high peroxidase activity in *ohy1* plants was not significantly reduced (**Figure 5C**). However, the exact mechanism by which ABA prevents ROS formation remains unclear.

Based on our findings, we propose a simple model (**Figure 6**) in which increased cuticle permeability results from ROS accumulation generated by apoplastic CIII peroxidases. ABA appears to attenuate ROS formation and promote the expression of genes involved in cuticle biosynthesis. In plants that exhibit the constitutively enhanced expression of peroxidases, the activation of ABA signaling, which can be triggered by factors including abiotic stress, was insufficient to completely remove excessive levels of ROS. This led to an increase in the permeability of the cuticle and primed plant defenses to necrotrophic pathogens. In the case of necrotrophic pathogens, when a plant recognizes cell wall-derived DAMPs, such as OGs, it activates a set of CIII peroxidases that redundantly generate ROS to promote resistance. However, the production of ABA by *B. cinerea* (Kitagawa et al., 1995; Siewers et al., 2006) might suppress this increase in the accumulation of ROS that is initiated by pathogen recognition, allowing only the transient production of ROS, resulting in overall decreased defenses and colonization by brute force necrotrophs. Therefore, the overexpression of any of these CIII peroxidases is sufficient to overcome the *in vivo* ABA-mediated removal of ROS, while lacking a single peroxidase isoform has no impact on a plant’s resistance phenotype.

Our results clearly indicate that peroxidase-generated ROS trigger strong repression of *MYB96, BDG* and *LACS2.3* transcripts, involved in cuticle biosynthesis and promote cuticular permeability. Because the enhanced accumulation of ROS was previously reported in the cuticular *bdg* and *lacs2.3* mutants (L’Haridon et al., 2011), this role for peroxidase-generated ROS suggests the existence of a positive feedback loop between ROS and the integrity of the cuticle. Our data also demonstrates that ABA signaling is significantly involved in attenuating the excessive formation of ROS, and this process is partially mediated by the down-regulation of peroxidase activity. Overall, the increased permeability of the cuticle leads to the activation of DAMP-responses through the primed expression of genes associated with OG-dependent defenses independently of the SA and JA signaling pathways. This ultimately increases resistance against necrotrophic bacterial and fungal pathogens.

## Author Contributions

MS, PD, VP, PH, and EP planned and designed the study. MS, PD, VP, MB, and NS performed the experiments and analyzed the data. MS, TK, PD, and PH wrote the manuscript.

## Conflict of Interest Statement

The authors declare that the research was conducted in the absence of any commercial or financial relationships that could be construed as a potential conflict of interest.

The reviewer VP and handling Editor declared their shared affiliation, and the handling Editor states that the process nevertheless met the standards of a fair and objective review.

## Footnotes

1 https://fiji.sc/

2 https://fiji.sc/
