# Supplementary material for: Peroxidase-Generated Apoplastic ROS Impair Cuticle Integrity and Contribute to DAMP-Elicited Defenses
Source: Front Plant Sci. 2016 Dec 23;7:1945. doi: 10.3389/fpls.2016.01945 (PMC5179520; doi:10.3389/fpls.2016.01945)
Supplement: Supplementary file 1 [file Table_1.PDF]

Table S1. Primers used in this study

Gene	AGI code	Forward primer	Reverse primer
		**for quantitative RT-PCR**	
UBQ10	AT4G05320	TCCAGGACAAGGAGGTATTCCTCCG	CCACCAAAGTTTTACATGAAACGAA
EF1α	AT5G60390	TCCAGCTAAGGGTGCC	GGTGGGTACTCGGAGA
PER57	AT5G17820	TGACAGAATCACTAGCTTCCA	TCAATGGACTCGACTGGTCT
PAD3	AT3G26830	ACGAGCATCTTAAGCCTGGA	TCGGTCATTCCCCATAGTGT
PER4	AT1G14540	GGAACCGGAGCTTCTACAGATAG	TTAATTAACGGCACTGCAGATTC
PGIP1	AT5G06860	GACGAATCTGACAGGTCCAA	ATAGGCGAAGGTCAGGGACT
GST1	AT1G02930	CAAGGACATGGCGATCATAGC	TCCCAAAGCTTTGAACCA
PR1	AT2G14610	CGGAGCTACGCAGAACAACT	CTCGCTAACCCACATGTTCA
PR5	AT1G75040	ATCACCCACAGCACAGAGACAC	AGCAATGCCGCTTGTGATGAAC
SID2	AT1G74710	GCGAGGAGAGTGAATTTGCAGTCG	CCACTCTGAAGATGGGTCACTTCCA
VSP2	AT5G24770	GGACTTGCCCTAAAGAACGACACC	GTCGGTCTTCTCTGTTCCGTATCC
CER1	AT1G02205	CAGGAACGGAGAGGTGTATATCCACAACCAT	CTATCAATGCTGGTGTGGTATGATAGATAC
WAX2	AT5G57800	AAGCATCCTGACCTTAGAGTTCGTGTGGTTCAT	TAAGACCATACTTCATGGCTGCTTCCCACA
LTP3	AT5G59320	GCAGGTAGCTTGCTCCATGTGCAAC	TATTTTATTCTAGTACTTCTGGTAA
LTP4	AT5G59310	GTGGCACAGTGGCAAGTAGCTTGAG	GATAGCCGTCTTATTTTACGTATACG
KCS2	AT1G04220	TCTCTTCGCCGCTCTTCTTATCTT	AGCGGTCGGAGGAGCGGTTAG

Gene	AGI code	Forward primer	Reverse primer
BDG	AT1G64670	TTCTTGGCTTTCCTCTTCCA	CCATAACCCAACAGGTCCAC
LACS2.3	AT1G49430	GTGCCGAGAGGAGAGATTTG	CGAGGTTTTCAACAGCAACA
PER28	AT3G03670	ACTATTCAAACGTCAGTTCGCA	TGAATGCTCTACAATTCGTCCT
PER34	AT3G49120	TAGGGTCGGGTAAACCTGTG	GTTGCTCTCTCCGGTGGTC
PER62	AT5G39580	ATGTCCAGTTCGCAAGGTCA	TAACCGCAGAGCAAACCCT
PER71	AT5G64120	ACAATTGATGGCTCCTAGAAGCA	TAACCGCAGAGCAAACCCT

for PCR cloning

PER7	AT1G30870	ATAGGTACCATGAAGTTGGCCGTGGTCT	AGAGCTCTTAGTAAGCTCTGGAGTTAGATTTGC
PER10	AT1G49570	ATAGGTACCATGGACCACAAAATGTCAATGT	AGAGCTCTTAACCTGGAAATCCACATTTTC
PER23	AT2G38390	TTGGCGCGCCATGGGGTTTTCGTCTTCATTATC	AGAGCTCTCAGATAGAACTCACAACACCATC
PER28	AT3G03670	ATGGCGCGCCATGAAGATTGCAACGTTTTCTG	TACTAGTTTAGTTGAATGCTCTACAATTCG
PER34	AT3G49120	ATAGGTACCATGCATTTCTCTTCGTCTTCAA	AGAGCTCTCACATAGAGCTAACAAAGTCAACG
PER44	AT4G26010	ATGGCGCGCCATGAGGTCAATCACAGCTTTG	AAAGAGCTCTCAGTTGTTGAAGACTCTGCA
PER53	AT5G06720	ATAGGTACCATGGCTGTAACAAATCTTCCTACT	AGAGCTCTCAACTTCCATTAACCTTCTTACAG
PER57	AT5G17820	AAAGGTACCATGATGAAGGGTGCCAAGT	AAAGAGCTCTTAGTTGAATCTCCTGCAGTTC
PER57	AT5G17820	AAAAAGCAGGCTCATCTCTTGTGAACGTTCCA	AGAAAGCTGGGTCAGATTGCGTACGATAGTCTC
PER64	AT5G42180	ATAGGTACCATGAATGCACACATGCTCAAT	AGAGCTCCTAGCGAACCCTTCTGCAGT

Note: Gene specific priemers for *CER1, WAX2, LTP3, LTP4, KCS2* were from [1], *EF1α* from [2], *VSP2* from [3], *PR1*, *AtPGIP1*, *PAD3*, *PER4* from [4].

1. Voisin D, Nawrath C, Kurdyukov S, Franke RB, Reina-Pinto JJ, Efremova N, et al. Dissection of the Complex Phenotype in Cuticular Mutants of Arabidopsis Reveals a Role of SERRATE as a Mediator. PLoS Genet. 2009;5: e1000703. doi:10.1371/journal.pgen.1000703.

2. Gutierrez L, Mauriat M, Guénin S, Pelloux J, Lefebvre J-F, Louvet R, et al. The lack of a systematic validation of reference genes: a serious pitfall undervalued in reverse transcription-polymerase chain reaction (RT-PCR) analysis in plants. Plant Biotechnol J. 2008;6: 609–618. doi:10.1111/j.1467-7652.2008.00346.x

3. Brotman Y, Riewe D, Lisec J, Meyer RC, Willmitzer L, Atlmann T. Identification of enzymatic and regulatory genes of plant metabolism through QTL analysis in Arabidopsis. J Plany Physiol 2011;168(12):1387-94. doi: 10.1016/j.jplph.2011.03.008.

4. Denoux C, Galletti R, Mammarella N, Gopalan S, Werck D, Lorenzo GD, et al. Activation of Defense Response Pathways by OGs and Flg22 Elicitors in Arabidopsis Seedlings. Mol Plant. 2008;1: 423–445. doi:10.1093/mp/ssn019
