# Supplementary material for: Peroxidase-Generated Apoplastic ROS Impair Cuticle Integrity and Contribute to DAMP-Elicited Defenses
Source: Front Plant Sci. 2016 Dec 23;7:1945. doi: 10.3389/fpls.2016.01945 (PMC5179520; doi:10.3389/fpls.2016.01945)
Supplement: Supplementary file 2 [file Table_2.PDF]

Table S2. List of *A. thaliana* OG-responsive class III peroxidases

No.	ID	Name	Fold change
1	AT1G14550	*PER5*	46.91877544
2	AT5G39580	*PER62*	33.20280705
3	AT1G14540	*PER4*	28.76005393
4	AT3G03670	*PER28*	25.39672468
5	AT5G05340	*PER52*	19.38386894
6	AT5G64120	*PER71*	17.19792933
7	AT5G64100	*PER69*	6.401402269
8	AT4G37530	*PER51*	6.077734028
9	AT4G08780	*PER38*	4.277084294
10	AT4G37520	*PER50*	4.131007162
11	AT5G64110	*PER70*	4.11715489
12	AT4G08770	*PER37*	3.090197406
13	AT3G49110	*PER33*	2.409877998
14	AT4G33420	*PER47*	2.407731621
15	AT1G49570	*PER10*	2.268852244
16	AT3G49120	*PER34*	1.968544835
17	AT4G36430	*PER49*	1.810347276
18	AT5G19890	*PER59*	1.794688383
19	AT3G01190	*PER27*	1.670244746
