# Supplementary figures and images for: Peroxidase-Generated Apoplastic ROS Impair Cuticle Integrity and Contribute to DAMP-Elicited Defenses
Source: Front Plant Sci. 2016 Dec 23;7:1945. doi: 10.3389/fpls.2016.01945 (PMC5179520; doi:10.3389/fpls.2016.01945)

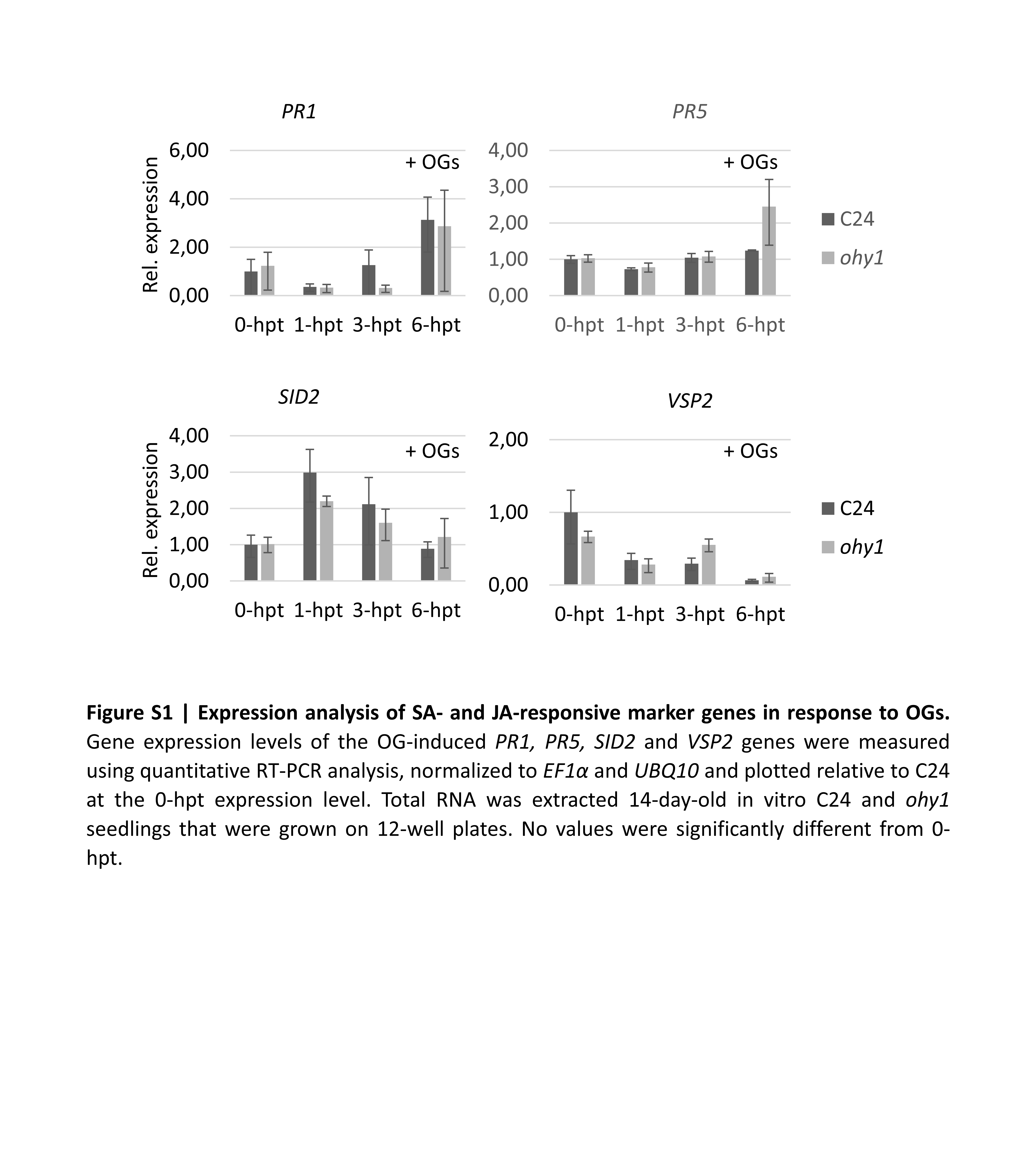

Supplement: Supplementary file 3 [file Image_1.TIF]

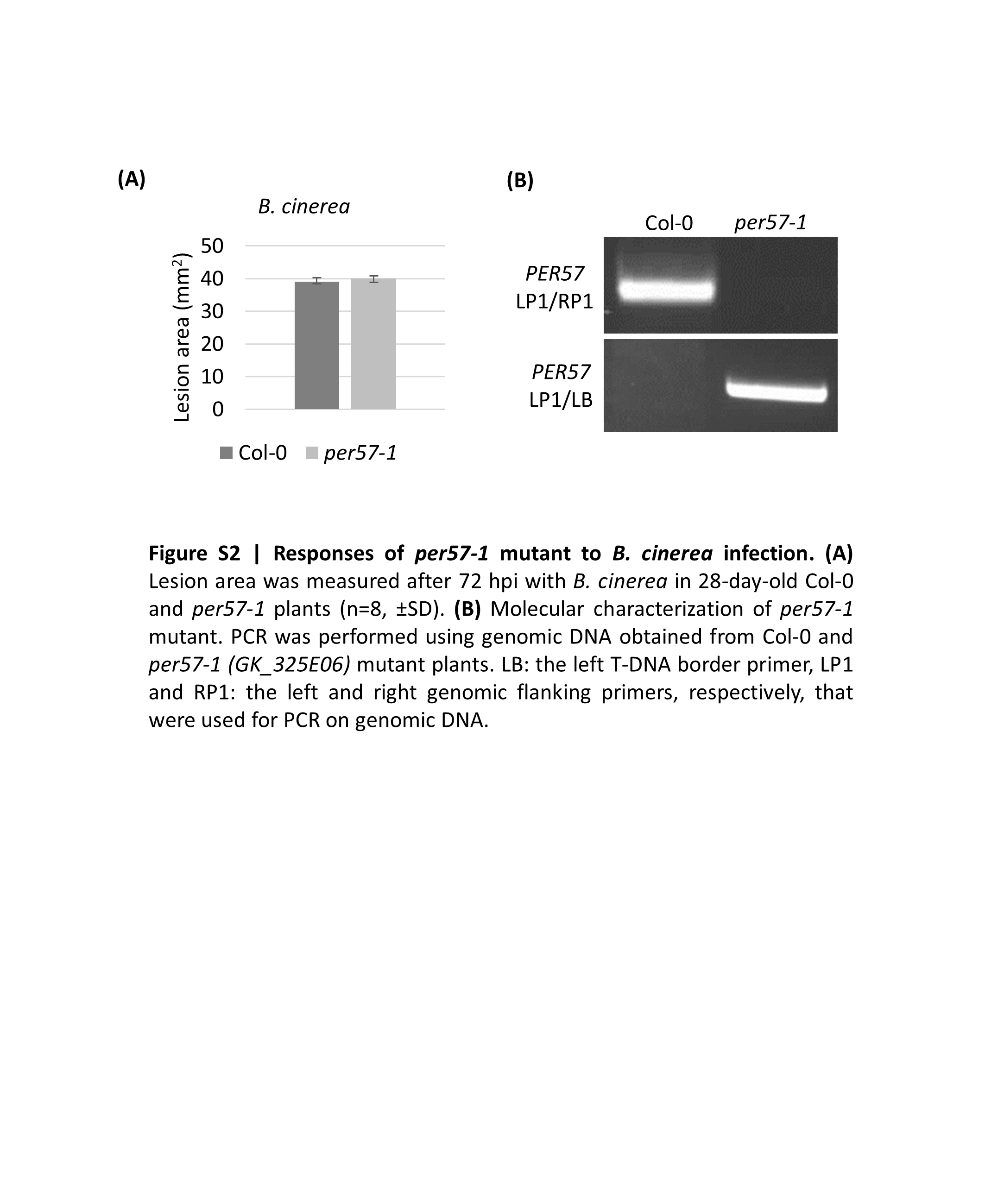

Supplement: Supplementary file 4 [file Image_2.TIF]

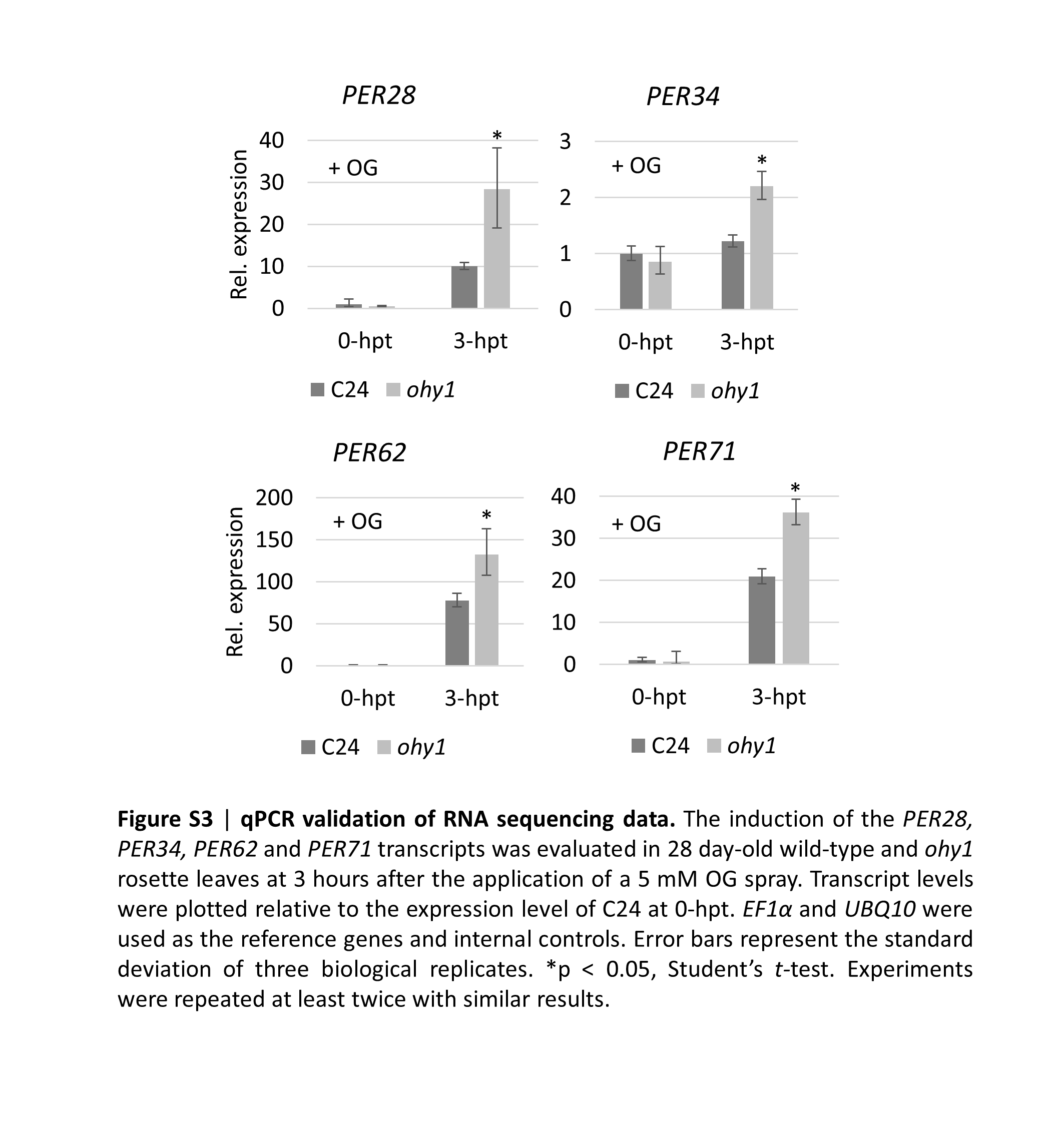

Supplement: Supplementary file 5 [file Image_3.TIF]

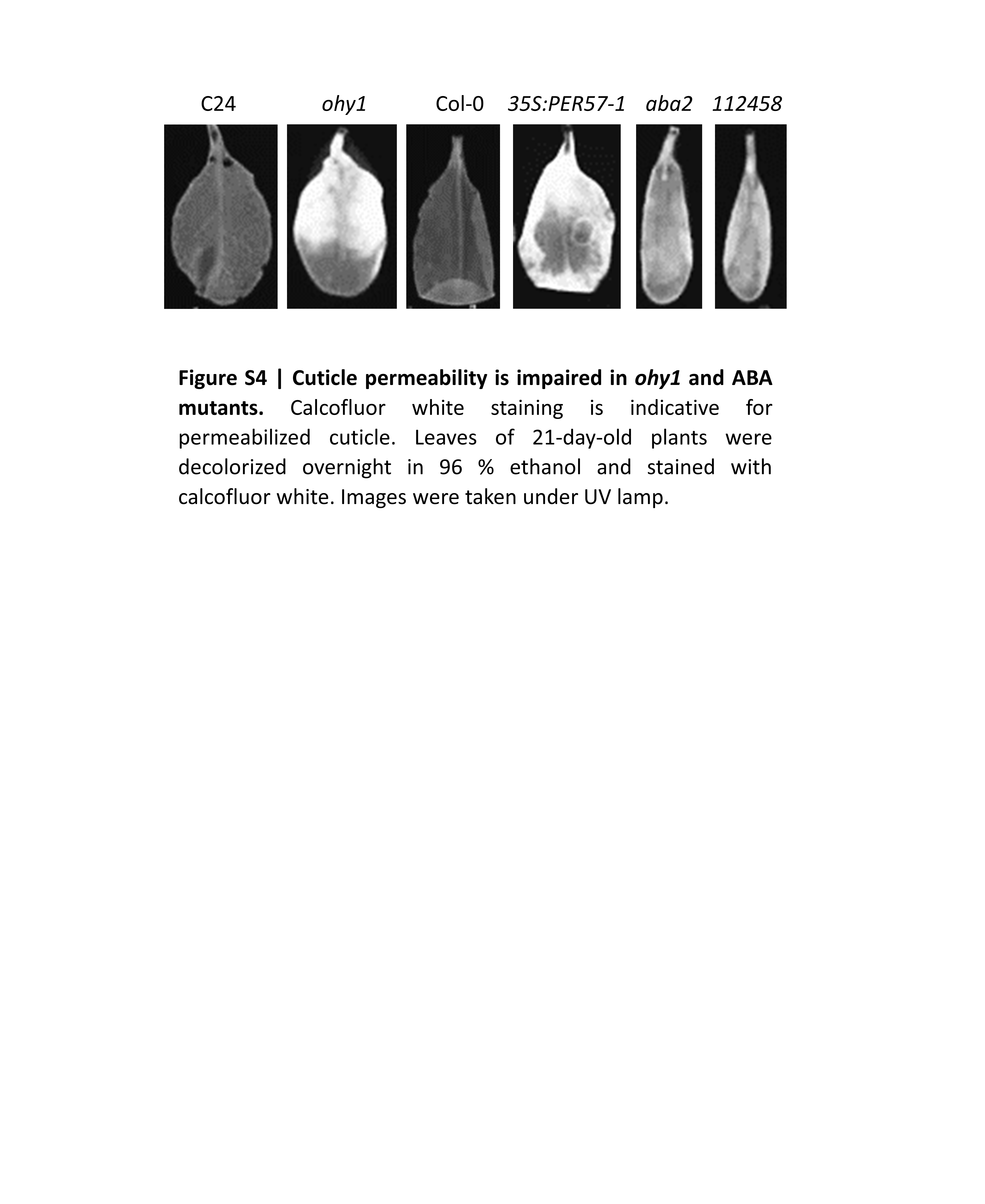

Supplement: Supplementary file 6 [file Image_4.TIF]

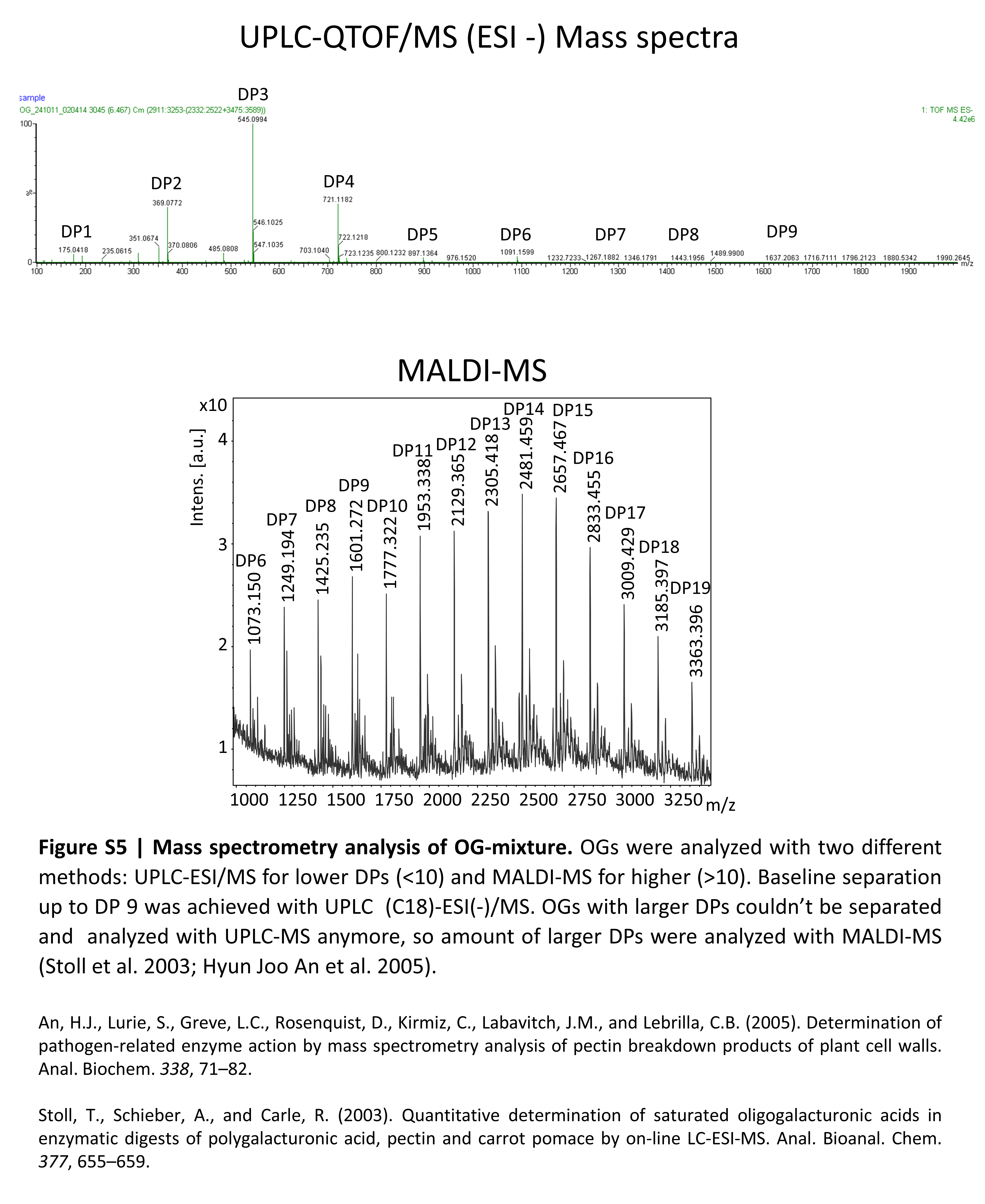

Supplement: Supplementary file 7 [file Image_5.TIF]
